# Large-scale Investigations of Water Diffusion in Metal–Organic Frameworks with One-Dimensional Channels

**DOI:** 10.1021/acsami.5c12959

**Published:** 2025-09-03

**Authors:** I-Ting Sung, Li-Chiang Lin

**Affiliations:** † Department of Chemical Engineering, 33561 National Taiwan University, No. 1, Sec. 4, Roosevelt Road, Taipei 106319, Taiwan; ‡ William G. Lowrie Department of Chemical and Biomolecular Engineering, The Ohio State University, 151 W. Woodruff Avenue, Columbus, Ohio 43210, United States

**Keywords:** water diffusion, metal−organic frameworks, large-scale computations, molecular simulations, structure−property relationships

## Abstract

Metal–organic frameworks (MOFs), known for their highly versatile nature, show considerable promise as adsorbents and membranes for water-related applications such as water harvesting and water filtration. One of the key factors that may influence their efficiency is the diffusion of water within MOFs. However, the behaviors and mechanisms of water diffusion in MOFs remain relatively underexplored. To this end, this study utilizes molecular dynamics (MD) simulations to comprehensively analyze water diffusion in hundreds of distinct MOFs. Herien, MOFs that exhibit rapid water diffusion under saturated conditions are identified. Moreover, structure–property relationships and underlying atomistic mechanisms are explored, showing that water mobility depends on a subtle interplay between pore topology and host–guest interaction. For instance, MOFs with similarly small pores can exhibit markedly different water diffusivities. In hydrophobic MOFs, weak framework-water interactions allow ultrafast single-file diffusion, but mobility plunges when the pores narrow further. In hydrophilic MOFs, strong framework-water interactions immobilize a layer of water on the pore walls; these fixed water molecules can interestingly shield strong adsorption sites, enhancing transport in wider pores yet impeding diffusion in narrower ones. Finally, to address the high computational cost associated with computing diffusivity, this work further evaluates the feasibility of using surrogate descriptors for rapid diffusivity estimation. Overall, this work offers molecular-level key insights into water transport phenomena.

## Graphical abstract

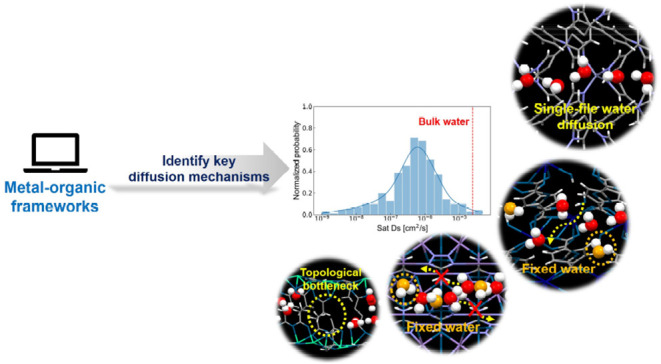

## 1 Introduction

With the continuous rise in global population and industrial activities, water demand has surged significantly. Currently, two-thirds of the world’s population resides in areas experiencing water scarcity, making it a major global risk that is expected to persist in the coming decades.^1^ To tackle this issue, sorption-based atmospheric water harvesting (AWH) has shown great potential, particularly in regions with low humidity.^2^ Another promising method to address water shortages is through water filtration applications. These include processes such as desalination, which removes salt ions from seawater, and wastewater treatment, which eliminates dissolved organic matter and ions from industrial wastewater to produce clean water.^3^ Recently, metal–organic frameworks (MOFs) have gained significant attention for their potential in these water-related applications.^4–7^ This is largely due to their versatile chemical and geometrical properties.^8^ MOFs are composed of metal nodes and organic linkers, allowing for adjustable hydrophilicity and topology by varying the metal-linker combinations.^9^ Given the large variety of MOFs, tens of thousands of distinct structures have been reported to date, and thus identifying the most promising ones can be quite challenging.^10^ To address this, recent advancements in molecular simulations have facilitated large-scale screenings of MOFs.^11,12^ Specifically, recent studies have been conducted to explore MOFs for optimal adsorption properties by utilizing high-throughput computational approaches. For instance, Sung et al. employed molecular simulations to identify optimal MOFs for CO_2_/CO separation applications.^11^

While the adsorption properties of MOFs are key to their potential in water-related applications, an equally important aspect is their water diffusion behavior. That is, in applications such as AWH, it is essential to identify materials that not only exhibit high water vapor sorption capacity but also enable efficient water diffusion.^13,14^ It has been reported that the productivity of advanced water adsorbents is often limited by slow internal water diffusion, which can hinder the practical application of these promising materials.^15^ Similarly, in the context of water filtration, membrane performance is heavily influenced by water diffusion behavior.^16^ By enhancing water transport, the permeability of water molecules through these membranes can be improved, resulting in greater efficiency.^17^ Despite its significance, water diffusion remains relatively underexplored compared to the transport of other gases such as CO_2_ and CH_4_.^18,19^

To this end, this study utilizes state-of-the-art molecular simulations to investigate water diffusion in MOFs on a large scale. The primary objectives are 2-fold: (1) to identify MOFs with ultrafast water diffusion and (2) to uncover diffusion mechanisms to inform future material design. The investigation begins with an extensive analysis of water diffusivity under saturated conditions (Sat Ds), observing a wide range of diffusivities, including instances of ultrafast diffusion. Subsequently, this study investigates water diffusion behaviors and summarizes key mechanisms. Besides, to accelerate future screening, diffusivity under dilute conditions (Dilute Ds) and free energy profiles are also examined as surrogates for Sat Ds. Overall, this work offers comprehensive insights into water diffusion within MOFs, identifying structure–diffusivity relationships that enable the design of MOFs with optimized water transport properties.

## 2 Computational Methods

This section begins by detailing the MOF structures considered in this study. Following this, the computational techniques utilized are presented, with MD simulations employed to analyze the self-diffusivity (Ds) of water within the MOFs and Monte Carlo (MC) simulations used to determine water uptakes and free energy profiles. Finally, the force field parameters applied to describe intermolecular interactions are introduced.

### 2.1 MOF Structures

In this work, MOF structures included in the 2019 Computation-Ready, Experimental (CoRE) MOF database^10^ are analyzed without modifications. Specifically, MOFs that have a pore limiting diameter (PLD) of at least 2.8 Å, corresponding to the kinetic diameter of a water molecule, are considered to ensure their water accessibility. The PLD values referenced herein are taken directly from the existing data in the 2019 CoRE MOF database.^10^ Besides, this study focuses only on orthorhombic MOFs with one-dimensional (1D) channels for a more straightforward comparison between the studied MOFs. From a pool of approximately 800 orthorhombic MOFs with 1D channels, considering the computational cost, we further randomly select 300 MOFs with diverse characteristics. Each MOF is characterized by complementary geometrical and chemical descriptors that together capture pore topology and its chemical properties. Geometrically, the PLD and the largest cavity diameter (LCD) quantify the narrowest bottleneck and the largest cavity along the diffusion path, with the former being considered as an indicator of the topological diffusion constraints. Other features, such as void fraction (θ), are also included to describe the structure. Chemically, because strong adsorption sites may slow water diffusion, descriptors that may reflect the site strength and density, including the most negative and maximum charges, the metal-to-oxygen ratio, and the metal-to-carbon ratio, are also included. Given that metal sites are usually the binding sites, we further assess metal site-related properties, such as metal angle and the metal dipole moment, to gauge the accessibility and charge separation of a certain metal. Together, these complementary descriptors provide an integrated picture of the framework. Figure 1 presents an overlay plot that compares the distribution of these geometric and chemical features among the studied 300 MOFs and the full set of orthorhombic MOFs with 1D channels. This plot clearly demonstrates that the studied MOFs are sufficiently representative; their feature distribution encompasses and aligns with that of the entire subset of MOFs. That is, the findings from this smaller subset may be generalized to infer the properties of all orthorhombic MOFs with 1D channels.

**Figure 1:**
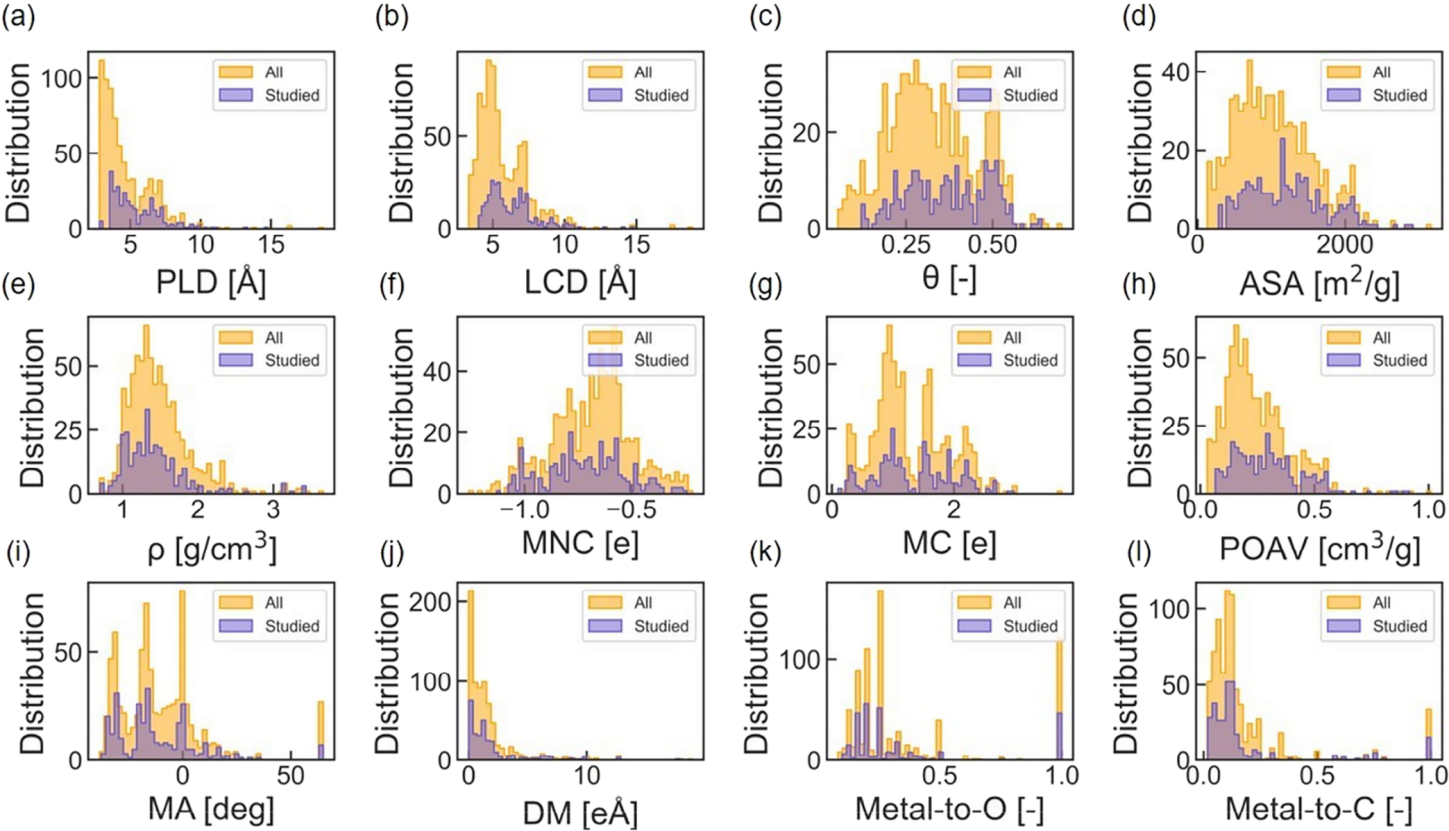
Overlay plot comparing the (a–e) geometrical and (f–l) chemical feature distribution of the studied MOFs, orthorhombic MOFs with 1D channels. The features considered herein include: (a) PLD, (b) LCD, (c) θ, (d) accessible surface area (ASA), (e) density (ρ), (f) most negative charge (MNC), (g) maximum charge (MC), (h) probe occupiable and accessible pore volume (POAV), (i) metal angle (MA), (j) DM, (k) metal-to-oxygen ratio (Metal-to-O), and (l) metal-to-carbon ratio (Metal-to-C). The geometric descriptors, i.e., POAV, θ, ASA, and ρ, are calculated with the open-source Zeo++,^20^ using a 1.32 Å helium probe and 50000 Monte Carlo insertion moves for each MOF. Chemical descriptors such as the metal-to-oxygen and metal-to-carbon ratios, the MNC and MC, are extracted directly from the crystallographic information files, while an in-house script^21^ is used to determine MA and the DM.

### 2.2 Molecular Dynamics (MD) Simulations for Diffusion Properties

The Ds of water in MOFs is determined through MD simulations conducted using the LAMMPS software.^22^ The simulations are performed in a canonical ensemble (i.e., constant molecular number, volume, and temperature (NVT)) at a temperature of 298 K modulated by the Nosé-Hoover thermostat with a damping factor of 100 time steps (i.e., 100 fs). We investigate water diffusivity under both saturated and dilute conditions. Here, the saturation loading refers to the water uptake at 298 K and 100% relative humidity, while the dilute loading is defined as 10% of the saturation loading. It is noted that the saturation loadings are determined by MC simulations, which will be detailed in the following section. The structure of all studied MOFs is kept fixed, and rigid water molecules are constrained using the SHAKE algorithm.^23^ It is noted that despite omitting the effect of structural flexibility, this choice is anticipated to still support meaningful, relative comparisons across MOFs. All MD simulations are conducted for a minimum duration of 50 ns, and the collected molecular trajectories are analyzed to calculate the mean squared displacement (MSD) using an in-house script. Subsequently, the Ds is determined from the slopes of ln­(MSD) versus ln­(time) with the Einstein relation from the segment where the slope is between 0.95 and 1.05 and is closest to one.

### 2.3 Molecular Carlo (MC) Simulations for Adsorption Properties

#### 2.3.1 MC Simulations for Saturation Loadings

Grand canonical Monte Carlo (GCMC) simulations implemented in the RASPA software^24^ are conducted at 298 K and 986 Pa, the saturation pressure for the TIP4P-EW water model,^25^ to determine the saturation loading of water. These conditions are used to calculate the saturated diffusion coefficient, referred to as Sat Ds. We note that, to mitigate potential convergence issues often encountered in GCMC methods for water adsorption,^25,26^ the so-called desorption simulations are carried out. That is, the simulation begins from saturation conditions, where the initial saturation loading is estimated by multiplying the free pore volume of the MOF by the density of liquid water. Before running the GCMC desorption simulations, the system is preliminarily relaxed in a canonical (NVT) ensemble at 298 K. In the NVT simulation, translation, rotation, and reinsertion moves are applied in a 1:1:1 ratio. The configuration resulting from this NVT simulation then serves as the starting point for the GCMC desorption simulations. During the GCMC simulations, millions of Monte Carlo moves, including translation, rotation, reinsertion, and swap moves in a 1:1:1:4 ratio, are performed to ensure the accuracy of the results.

#### 2.3.2 Heats of Adsorption (HOA)

The heat of adsorption (HOA) at infinite dilution effectively represents the framework-water interactions, serving as an efficient metric for evaluating the sorption nature of a material.^27^ Typically, more negative HOA values correspond to stronger framework-water interactions, reflecting a more hydrophilic character, whereas more positive values indicate weaker interactions, indicating a more hydrophobic nature.^28^ To calculate HOA, the Widom particle insertion method, also implemented in the RASPA software,^24^ is used, with each simulation running for 100,000 cycles at 298 K. To ensure accuracy, simulations are conducted with a criterion that the standard deviation of the obtained HOA must not exceed 10% of the mean value. If this criterion is not met, the simulations are extended by another 100,000 cycles. Due to computational costs, a maximum of 500,000 cycles per MOF is set.

### 2.4 Intermolecular Interactions

For both MD and MC simulations, Lennard-Jones (L-J) and electrostatic interaction energies are included to describe intermolecular interactions. L-J parameters for MOF atoms are derived from the DREIDING^29^ and the universal force field (UFF)^30^ systems, with UFF parameters specifically applied to elements not covered by DREIDING. Atomic charges for MOFs are determined using the multilayer connectivity-based atom contribution (m-CBAC) method,^31^ based on training data from high-quality charges computed via density functional theory (DFT).^32^ It is noted that to enable large-scale comparisons with affordable computational costs, charges are treated as fixed, which do not explicitly account for charge transfer or polarization. A subset of materials can subsequently be studied using higher-level approaches. Since the TIP4P-Ew water model consistently reproduces experimental bulk water diffusivity, we therefore employ this model for water in our simulations.^33^ Besides, prior work using the same simulation setup, which is UFF/Dreiding force field combined with TIP4P-Ew water model, has reported adsorption isotherms and heats of adsorption values for MIL-100­(Fe) that closely match experimental data. The authors also note that experimental water diffusivity data for MIL-100­(Fe) were lacking; nonetheless, their predicted value (∼4.8 × 10^–10^ m^2^/s at full saturation) aligned with literature ranges for water in other MOFs.^34^ It is noted that although this MOF is not in our studied database, our diffusivity results are also consistent with this magnitude. Importantly, we note that direct experiment-simulation comparisons for water mobility in MOFs are inherently challenging because transport properties are highly sensitive to material-specific details that are often under-reported. In particular, variations in defect types/densities (e.g., missing linkers) can markedly alter hydrophilicity, and differences in activation protocols can leave residual coordinated water. These widely noted factors can shift measured properties by orders of magnitude, complicating one-to-one benchmarking.^35,36^ Details on the force field parameters and the partial charges for the water model are available in the Supporting Information (SI). The Lorenz-Berthelot mixing rule is applied for interactions involving different elements, and L-J potentials are truncated and shifted at a 12 Å cutoff distance. For long-range interactions, the particle–particle-particle-mesh (PPPM) technique and the Ewald summation method are used, respectively, in MD and MC calculations.

## 3 Results and Discussion

In this section, this study begins by showing the large-scale Sat Ds results of water diffusivity for 300 MOFs, followed by exploring the relationship between the Sat Ds and the characteristics of MOFs. The discussion next clarifies the principal water-diffusion mechanisms, emphasizing the effect of strongly adsorbed water (i.e., fixed water) uncovered in this study. Insights from these mechanisms then guide engineering strategies that tune water adsorption strength while simultaneously promoting rapid diffusion within MOFs. Given the high computational cost of obtaining Sat Ds, we also assess whether diffusivities under dilute conditions (Dilute Ds) can inform Sat Ds. A discussion on employing free energy landscapes to infer diffusivities is also offered.

### 3.1 Large-Scale Study on Water Diffusion in MOFs

The large-scale computation results, as illustrated in Figure 2, interestingly reveal a broad distribution of the diffusivity of water in MOFs under saturated conditions, ranging from as restricted as 10^–9^ [cm^2^/s] to as fast as 10^–5^ [cm^2^/s]. The diffusivity can vary over 4 orders of magnitude as a result of their diverse structural topologies and chemical properties. Notably, it appears that most MOFs exhibit Sat Ds around 10^–6^ cm^2^/s, with an average value of 1.81 × 10^–6^ [cm^2^/s] and a standard deviation of 5.02 × 10^–6^ [cm^2^/s]. Water diffusion in MOFs has remained rarely reported, with reported values generally ranging from as high as 10^–5^ [cm^2^/s] to as low as 10^–8^ [cm^2^/s]. For example, Salles et al. conducted MD simulations at 300 K and demonstrated that the flexible MIL-53 (Cr) exhibited different Ds values in its narrow pore (NP) and large pore (LP) forms. Their results indicated that, across a range of water loadings, water diffusion is consistently faster in the LP form. Specifically, the Ds values for the NP form ranged from 8 × 10^–6^ to 1.5 × 10^–7^ cm^2^/s, while those for the LP form span from 10^–5^ to 2.5 × 10^–7^ cm^2^/s, respectively.^37^ Similarly, Li et al.^38^ performed MD simulations at 300 K to study water diffusion under varying water loadings. Their findings showed that the fastest water diffusion occurred in proline-Ni-CPO-54, a water-stable MOF, with a self-diffusion coefficient on the order of 10^–8^ [cm^2^/s]. Experimentally, water self-diffusivity in UTSA-280 is on the order of 10^–8^ cm^2^/s,^39^ and for aluminum fumarate it spans 10^–6^–10^–8^ cm^2^/s.^40^ Although the study of water diffusion in MOFs is still limited, existing reports on the range of water diffusivities consistently align with our findings. Importantly, with the outcomes of the large-scale MD simulations conducted in this study, a comprehensive large-scale analysis of water diffusion in MOFs is reported herein for the first time.

**Figure 2:**
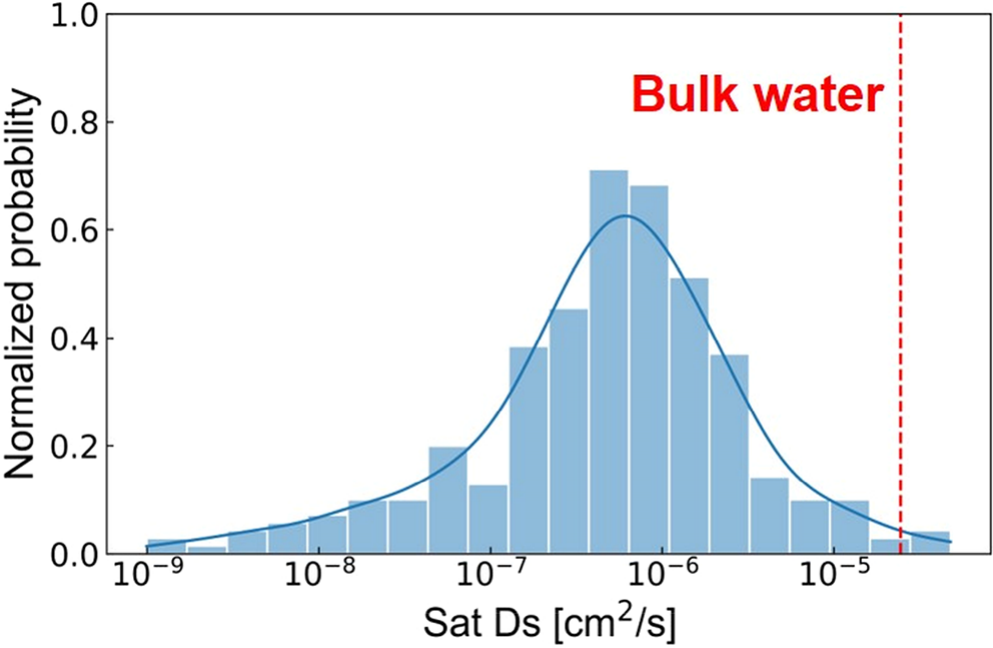
Normalized probability distribution of MD-computed water diffusion coefficient under saturated conditions (Sat Ds).

Interestingly, several MOFs in this work are predicted to have water diffusivities that even exceed those of both bulk water (i.e., calculated as 2.44 × 10^–5^ [cm^2^/s] at 298 K with the molecular potential employed in this study, while the experimentally measured value at room temperature is 2.3 × 10^–5^ [cm^2^/s][Bibr ref41]) and even carbon nanotubes (CNTs), which are well-known for facilitating rapid water diffusion.^42^ It is widely recognized that single-file diffusion occurs in CNTs with diameters smaller than 10 Å, forming a one-dimensional hydrogen bond network.^43–45^ Several studies, both theoretical and experimental, have explored water behavior in such CNTs.^45,46^ For example, Kalra et al. studied CNTs with a diameter of 8.1 Å, noting that water in these CNTs adopts a single-file arrangement with the entire water chain moving collectively via a 1D random walk.^46^ Similarly, Mukherjee et al. studied (6, 6) CNTs with diameters of 8 Å and varying lengths (i.e., 14, 28, and 56 Å) in a TIP3P water bath, observing diffusion coefficients of 2.2 × 10^–5^ cm^2^/s, 2.5 × 10^–5^ cm^2^/s, and 2.8 × 10^–5^ cm^2^/s, respectively. Their results showed that water molecules in these CNTs diffuse in a single-file manner.^47^ Notably, MOFs with similar characteristics to CNTs (i.e., narrow and hydrophobic 1-D channels), such as PEKZIP, NAVLIG, and OHIHET, show even greater Ds values of 4.08 × 10^–5^ cm^2^/s, 4.31 × 10^–5^ [cm^2^/s], and 4.78 × 10^–5^ [cm^2^/s], respectively. Their corresponding PLD values are 4.09, 3.74, and 3.71 Å, which are all below the data set average of 5.44 Å. This enhanced diffusivity may be attributed to differences in their HOA values (i.e., framework-water interactions). Specifically, the HOA values for these MOFs are 14.25, 17.66, and 14.96 kJ/mol, respectively, while the HOA value of the (6, 6) CNT is approximately 18.22 kJ/mol.^48^ The increased hydrophilicity associated with higher HOA values represents stronger water-MOF interactions, which slow down the water diffusion rate as the greater attractive force causes water molecules to experience temporary retention along the channel walls during diffusion. A similar trend is observed in certain MOFs, where water molecules align in a single-file arrangement yet exhibit slower diffusivity due to their higher HOA values. For example, LUFQUZ02, PEPKUR, FAJZOI, and DOYBUQ display lower diffusion coefficients of 4.99 × 10^–6^ cm^2^/s, 1.78 × 10^–6^ cm^2^/s, 2.92 × 10^–6^ cm^2^/s, and 8.07 × 10^–6^ cm^2^/s, respectively. These MOFs have higher HOA values of 22.89, 24.20, 34.48, and 23.34 kJ/mol, respectively.

With the large-scale results at our disposal, we next explore the relationship between the Sat Ds and some key properties of MOFs. In particular, we emphasize two critical characteristics: the pore limiting diameter (PLD) and the HOA calculated under dilute conditions. This work emphasizes these characteristics since PLD often acts as the topological diffusion bottleneck in MOFs, making it a nearly exclusive metric for assessing diffusion behaviors. While ASA and POAV are also useful geometrical descriptors, as compared to PLD, they provide only indirect indications of geometrical constraints. Therefore, they are not employed in defining the diffusion mechanisms. Meanwhile, HOA, determined under dilute conditions, as noted above, reflects the strength of framework-water interactions—a more negative HOA indicates stronger interactions, generally restricting water diffusion. Figure 3a shows a general positive correlation between Sat Ds and PLD; a greater aperture size allows a faster diffusion, as would be intuitively anticipated. The result also implies that smaller PLDs restrict water diffusion more significantly. Interestingly, some results appear counterintuitive. Despite having small PLD values—typically associated with more restricted diffusion—certain MOFs exhibit significantly different diffusion behaviors. Specifically, as discussed previously, hydrophobic MOFs with small PLDs, where the pore sizes are comparable to a single water molecule, demonstrate single-file mode diffusion. This unique diffusion mode allows water molecules to diffuse faster than expected, resulting in higher Sat Ds than bulk water, which contradicts the conventional expectation based on PLD size alone.

**Figure 3:**
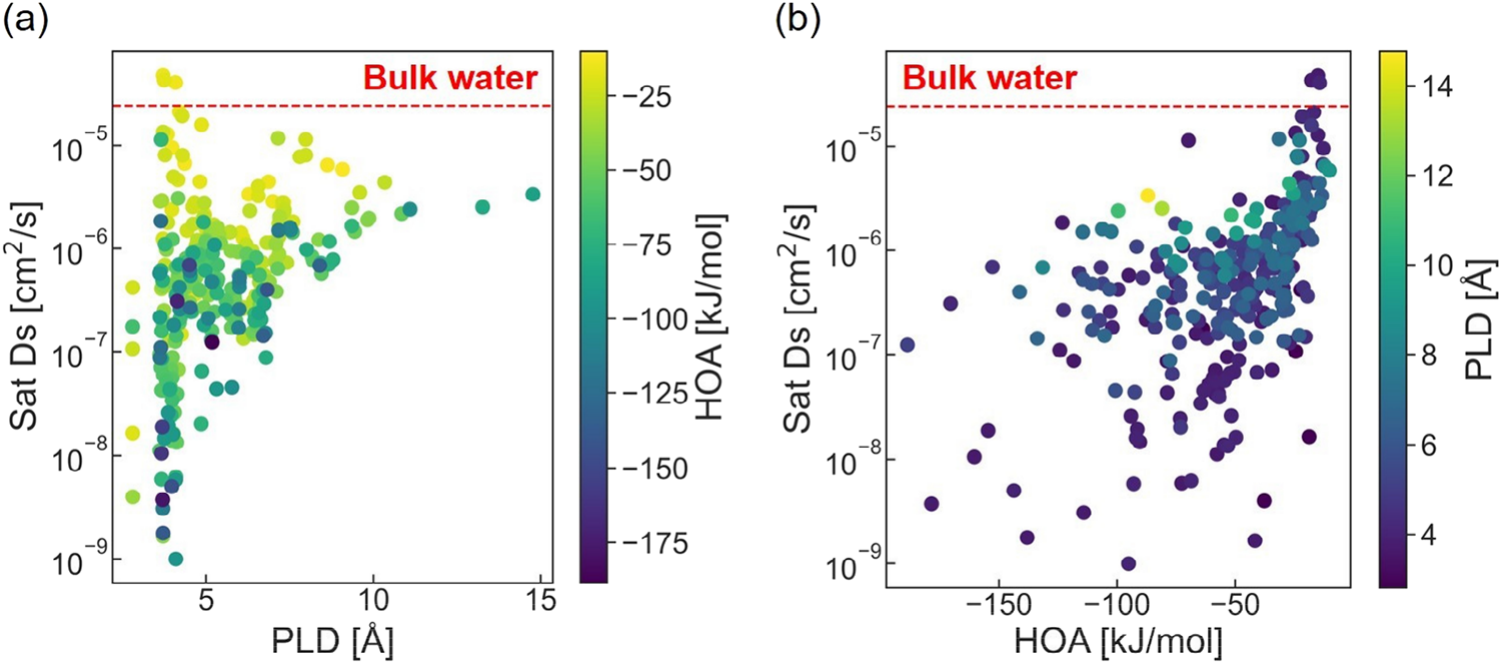
Relationships between (a) Sat Ds and PLD, color-coded by the HOA values, as well as (b) Sat Ds and HOA, color-coded by the PLD values. Warmer colors represent MOFs of less favorable HOA or greater PLD.

Figure 3b again illustrates that, consistent with prior studies, more positive HOA values correspond to faster Sat Ds. This observation may be attributed to the weaker framework-water interactions, associated with more positive HOA values, imposing fewer energetic constraints on water diffusion. However, MOFs with similar HOA values can again interestingly exhibit varying Sat Ds, suggesting that factors beyond energetic constraints may influence diffusion properties. Figure S1 further presents the correlations between Sat Ds and several other MOF features. For instance, Figure S1d,h, as expected, reveal that faster Sat Ds are associated with larger surface areas and pore volumes. Analogy to those MOFs of a greater PLD, under saturated conditions, these characteristics facilitate free exploration of the MOF structure by water molecules. Figure S1g,j indicate that faster Sat Ds are linked to smaller maximum metal charges and dipole moments. These features similarly result in weaker adsorption sites and a more homogeneous energy landscape. As a result, water molecules can traverse the structure more freely without being trapped at specific adsorption sites, leading to faster diffusion. Although each feature shows some correlation with Sat Ds, the relationships are highly nonlinear, and no single feature can fully capture diffusion behavior. A more rigorous approach would be to employ machine learning models that integrate multiple descriptors to account for these nonlinearities and provide insights through feature importance analysis.

Overall, the results align with our expectations: smaller PLD values hinder water diffusion under saturated conditions, and stronger host–guest interactions also reduce water diffusion. However, as mentioned earlier, some counterintuitive findings exist. Drastic variations in Sat Ds are observed among MOFs with small PLD values or similar HOA values, suggesting a complex interplay of energetic and topological factors. This highlights the need for further investigation into their diffusion mechanisms, which will be identified and discussed in section 3.2.

### 3.2 Key Water Diffusion Mechanisms

#### 3.2.1 Overview and Classification of Water Diffusion Mechanisms

Section 3.1 shows that MOFs with similar HOA or small PLD values display great variations in Sat Ds. After delving into this phenomenon, this work identifies the influence of structural bottleneck as has been widely accepted in the literature, the key role of water adsorption in governing overall water diffusion, and, more importantly, the interplay between these two factors, especially when strong adsorption sites are present in MOFs. Overall, we summarize four key diffusion mechanisms: 1. MOFs with large pores (i.e., PLD > 5 Å) and strong water adsorption (i.e., HOA < −60 kJ/mol) may exhibit a counterintuitively fast diffusion mechanism known as strong adsorption-shielded diffusion. 2. MOFs with small pores (i.e., 5 Å > PLD > 3 Å) and also strong adsorption (i.e., HOA < −60 kJ/mol) can also, by contrast, lead to a slow diffusion mechanism denoted as fixed water-hindered diffusion. 3. MOFs with unrestricted pores (i.e., PLD > 3 Å) and weak adsorption (i.e., HOA > −40 kJ/mol) enable a diffusion mechanism referred to as unconstrained water diffusion. 4. MOFs with restricted pores (i.e., 3 Å > PLD > 2.8 Å) result in a diffusion mechanism termed topological bottleneck-hindered diffusion. It should be noted that the cutoff value used to differentiate each mechanism is an approximate estimate. That is, although the kinetic diameter of water is 2.8 Å, implying that two layers would ideally need a 5.6 Å channel, this value is a simplification based on a spherical model. Since our simulations reveal the onset of the strong adsorption–shielded diffusion mechanism at PLD ∼ 5.25 Å, this work adopts PLD = 5 Å as a cutoff to distinguish the diffusion regimes. A schematic illustration of these four mechanisms can be seen in Figure 4.

**Figure 4:**
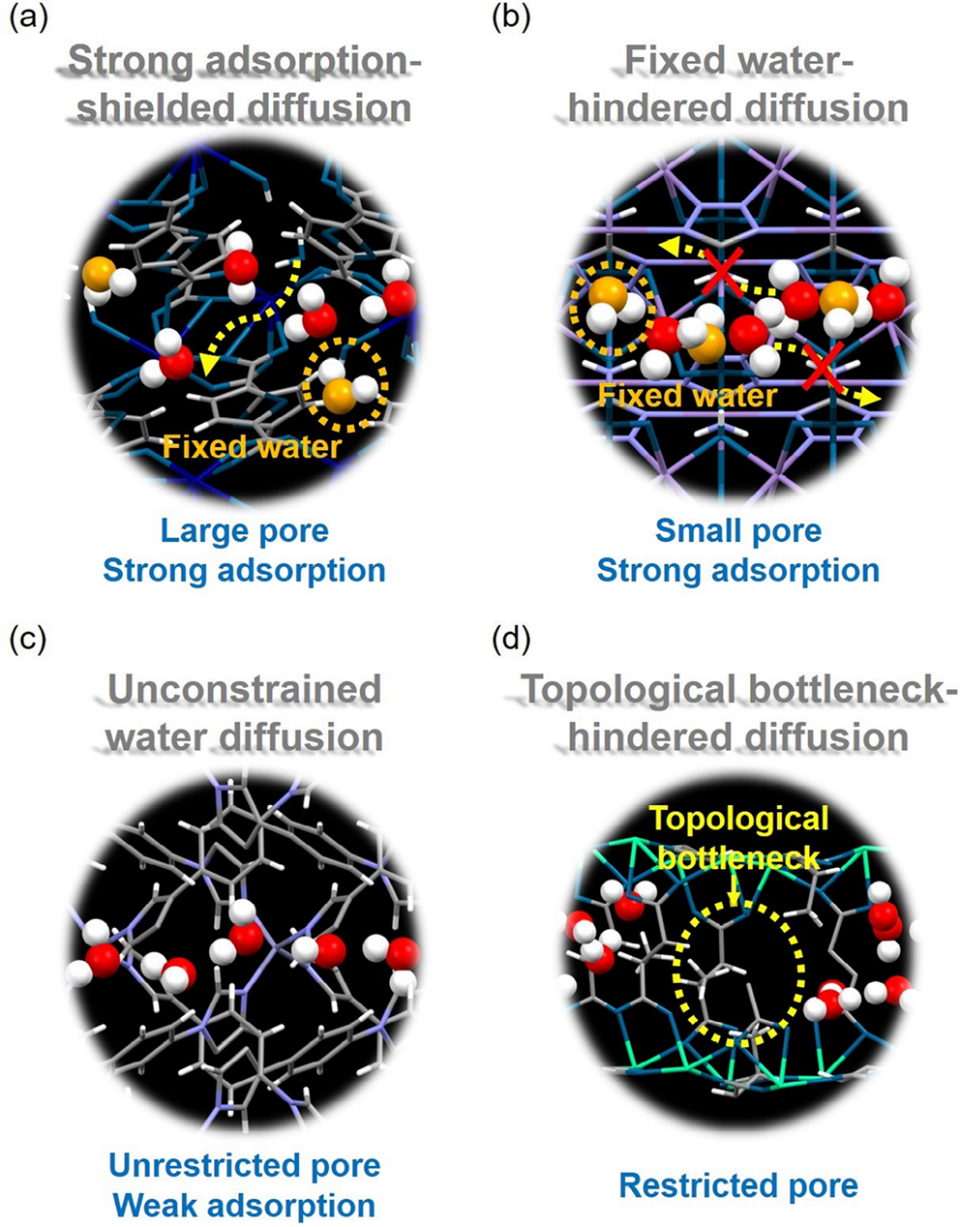
Key water diffusion mechanisms identified from the large-scale computational study, including (a) strong adsorption-shielded diffusion, (b) fixed water-hindered diffusion, (c) unconstrained water diffusion, and (d) topological bottleneck-hindered diffusion. In the upper two panels, fixed water molecules are colored in yellow, whereas free water molecules are shown in red.

#### 3.2.2 “Strong Adsorption-Shielded” and “Fixed Water-Hindered” Diffusion

MOFs with similarly strong adsorption strengths uncover intriguing phenomena. Specifically, for MOFs with strong water adsorption, dramatically different water diffusion behavior can be observed. MOFs herein have strongly adsorbed water molecules; upon adsorption in MOFs, these water molecules remain fixed and are referred to as “fixed water” hereafter. Fixed water can form at open metal sites or at highly hydrophilic functional groups (e.g., −OH), and its presence is reflected by a more negative HOA. For clarity, we illustrate this effect primarily at metal sites. To verify the existence of fixed water molecules, a case study is carried out to measure the metal-fixed water distance in FULQUZ from collected MD trajectories. As clearly shown in Figure S2, there exists a nearly constant metal-fixed water distance, recorded every 1000 time steps (1 ps). The average distance is found to be approximately 2.37 Å with a minimal standard deviation of 0.07 Å, confirming their immovable nature. Depending on the pore characteristics of MOFs, fixed water molecules interestingly influence diffusion in two distinct ways. In large-pore MOFs, although a monolayer of strongly adsorbed water molecules lines the framework walls, it effectively shields the strong adsorption sites. This shielding reduces the strength of subsequent framework-water interactions, facilitating efficient diffusion for water residing near the center of the pores. This phenomenon is referred to as the strong adsorption-shielded diffusion mechanism. In contrast, small-pore MOFs exhibit a different scenario. Here, the same monolayer of adsorbed water occupies a substantial fraction of the available pore space, acting as a steric barrier. Consequently, the limited remaining space severely restricts the diffusion of additional water molecules, a scenario described as the fixed water-hindered diffusion mechanism. Thus, pore size critically determines whether strongly adsorbed water molecules facilitate or hinder water diffusion in MOFs.

A schematic illustrating both diffusion mechanisms is presented in the upper left and right corners of Figure 4. Supporting movies provided in the SI further demonstrate these mechanisms. In these videos, frames are recorded every 10 time steps, with fixed water molecules highlighted in yellow. The fixed water-hindered diffusion mechanism, as shown in Figure 4 and more clearly from the corresponding SI Movie S1, reveals that freely diffusing water molecules become trapped between the fixed water molecules. In contrast, the strong adsorption-shielded diffusion mechanism, as observed in the respective SI Movie S2, shows that freely diffusing water molecules can diffuse more easily in the presence of fixed water molecules. For better visualization, one of these freely diffusing water molecules is labeled in green.

To better understand the shielding effect of fixed water, we use FULQUZ as a case study to examine its impact on water diffusion and free energy barriers. The shielding effect occurs once all strong adsorption sites are saturated, beyond which freely diffusing water molecules lead to a substantial increase in the Ds of water. Figure 5a shows the relationship between the number of water molecules per supercell (i.e., 1, 50, 96, 100, 250, and 500 #/supercell) and the corresponding Ds values. The Ds values for 1, 50, 96, 100, 250, and 500 water molecules are 2.33 × 10^–10^ cm^2^/s, 2.56 × 10^–10^ cm^2^/s, 2.87 × 10^–10^ cm^2^/s, 1.67 × 10^–6^ cm^2^/s, 4.77 × 10^–6^ cm^2^/s, and 1.09 × 10^–6^ cm^2^/s, respectively. These results indicate that in FULQUZ, which contains 96 metals providing a total of 96 strong adsorption sites, water diffusion remains relatively low (∼10^–10^ cm^2^/s) when the number of water molecules is 96 or fewer. However, once this threshold is surpassed, Ds increases dramatically to the order of 10^–6^ cm^2^/s or higher. As also reflected in the free energy plots with and without the shielding effect (Figure 5b), the presence of fixed water indeed significantly lowers the energy barrier experienced by freely diffusing water molecules. These confirm that the shielding effect of fixed water can notably enhance diffusion, specifically under conditions that are beyond the saturation point.

**Figure 5:**
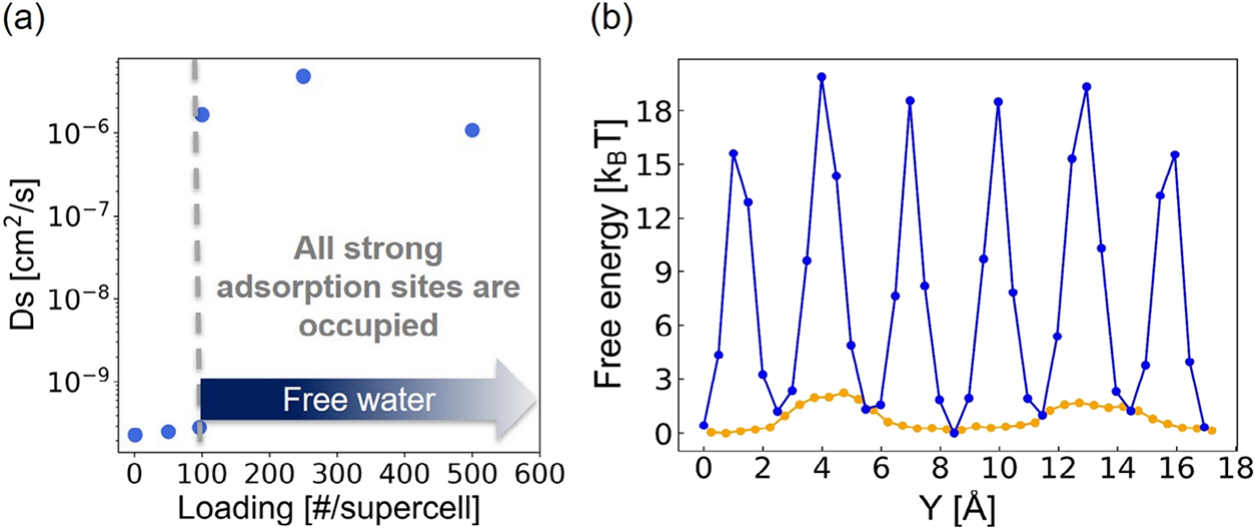
(a) The relationship between the number of water molecules (i.e., 1, 50, 96, 100, 250, and 500) and the corresponding self-diffusivity (Ds) of water in FULQUZ. (b) Comparison of 1D free energy profiles for water diffusion in FULQUZ with (orange line) and without (blue line) the shielding effect of fixed water. The barrier without shielding is computed using the Widom insertion method. With shielding, the barrier is obtained from NVT simulations at 298 K involving 97 water molecules, indicating that the shielding effect arises after the adsorption of 96 molecules. In this scenario, only one water molecule freely diffuses, experiencing the lowered energy barrier resulting from shielding.

#### 3.2.3 “Unconstrained Water” and “Topological Bottleneck-Hindered” Diffusion

In MOFs with weak adsorption sites, the metal-water interaction strength is not sufficiently strong to form fixed water, allowing water to diffuse through the channels. This is known as unconstrained water diffusion. This diffusion mechanism is illustrated in the lower left corner of Figure 4. Typically, the diffusion coefficient in this group is strongly correlated with the PLD. However, intriguing exceptions emerge—certain MOFs with smaller PLDs surprisingly exhibit ultrafast diffusion. In fact, as discussed previously, three MOFs in this category achieve Sat Ds values that can even surpass those of bulk water. These MOFs, characterized by small PLDs and hydrophobic channels, promote ultrafast single-file water diffusion. This rapid collective movement results from strong hydrogen bonding among water molecules, combined with minimal friction due to weak framework-water interactions.^49,50^ However, if the PLD of MOFs is comparable to the kinetic diameter of water molecules (i.e., 2.8 Å), topological bottleneck-hindered diffusion may then be observed. This mechanism is illustrated in the schematic at the lower right corner of Figure 4. As depicted in Figure S3a–c, a notably low water density is observed at the topological bottlenecks, evidently suggesting that the small pore aperture hinders water diffusion. However, in Figure S3d,e, the opposite trend is observed—high water density accumulates at similar bottlenecks, suggesting an alternative mechanism at play. This contrast can be attributed to the Coulombic effect,^51,52^ which can transform bottlenecks into preferential adsorption sites by reshaping the energy landscape of the diffusion channel. Depending on how the energy landscape is altered, the Coulombic effect can either promote or impede water diffusion at these critical sites. Nevertheless, even when it aids water transport, the inherently narrow bottleneck restricts water movement, ultimately slowing overall diffusion. A summary of the four types of diffusion mechanisms is provided in Table S3, which lists the MOF refcodes along with their chemical formula corresponding to each diffusion mechanism, along with their HOA and PLD values for a clearer comparison. Additionally, a comprehensive database detailing the diffusion properties and features of the studied MOFs is available in the SI for reference.

### 3.3 Surrogate Descriptors for Inferring Sat Ds

#### 3.3.1 Using Water Diffusivity under Dilute Conditions to Predict That under Saturated Conditions

While employing MD simulations represents a promising means to probe Ds, they can be costly. This section explores whether the more computationally efficient Dilute Ds can be used to infer Sat Ds. Figure 6a shows the distribution of the ratio of self-diffusivity in dilute conditions to that in saturated conditions (Dilute-to-sat Ds ratio). The results indicate that the Dilute-to-sat Ds ratio generally concentrates around 1, but it can vary significantly, ranging from as low as 10^–4^ cm^2^/s to as high as 10^2^ cm^2^/s. The Pearson correlation between log_10_(Sat Ds) and log_10_(Dilute Ds) is 0.535, indicating a moderate positive association. Correspondingly, Figure 6b shows an overall positive correlation between Dilute and Sat Ds. However, there is a significant number of outliers. These outliers share two decisive traits. First, their HOA values are more negative, which results in the formation of fixed water with a shielding effect. Second, Figure 6c demonstrates that these outliers display relatively large PLD values, ranging from about 5 to 8 Å. As noted in Section 3.2, MOFs with these characteristics correspond to the strong adsorption-shielded diffusion mechanism, i.e., under dilute conditions, fixed water molecules dominate the system, while under saturated conditions, they shield the strong adsorption sites and allow additional water molecules to diffuse freely, which then dominate the system. Interestingly, for MOFs with very large PLD values (i.e., >10 Å), the results for Dilute and Sat Ds are similar. This similarity arises because MOFs with very large PLD values exhibit higher water loadings. Under both dilute (i.e., 10% of the saturated loading) and saturated conditions, the strong adsorption sites may be saturated, resulting in the shielding effect of fixed water in both scenarios. Besides, large PLDs intrinsically impose fewer topological constraints on water diffusion.

**Figure 6:**
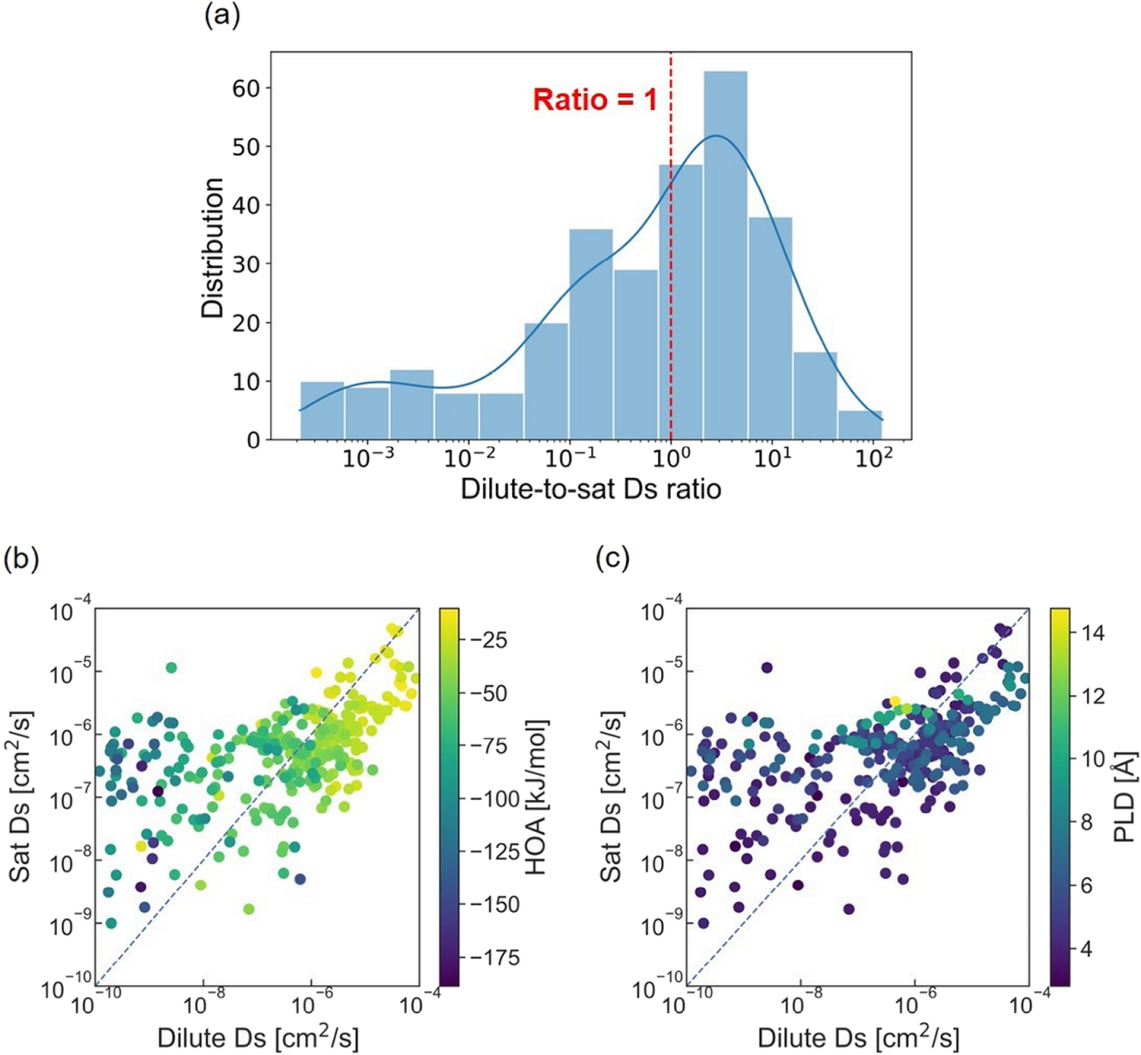
(a) The distribution of the ratio of Ds under dilute to saturated conditions (Dilute-to-sat Ds ratio). The ratio of 1 is indicated by the red dashed line in the plot. Correlations among diffusivity under saturated conditions (Sat Ds) and under dilute conditions (Dilute Ds), color-coded by (b) HOA and (c) PLD.

#### 3.3.2 Using Free Energy Profiles to Predict Water Diffusivities

To reduce the computational costs associated with directly calculating the Ds of water, another surrogate descriptor is free energy profiles. This section aims to investigate the feasibility of using the computationally efficient free energy profiles to accurately infer the Ds of water. Previous studies have widely employed 1D free energy plots for this purpose,^53^ as the barrier height in these plots allows for direct calculation of Ds using transition state theory.^54^ Herein, we explore the applicability of both 1D and 2D free energy profiles to infer water diffusivity under various conditions. Note that a detailed quantitative evaluation based on free energy profiles is not pursued herein, as such profiles are inherently high-dimensional. We begin by examining the 1D free energy profiles of several MOFs under dilute conditions. As illustrated in Figure 7a–h, MOFs that exhibit fast unconstrained water diffusion under these conditions generally show low 1D free energy barriers, typically smaller than 2 [*k*_B_ *T*]. Conversely, Figure 7i shows that MOFs like FULQUZ, which exhibit strong adsorption-shielded diffusion, exhibit slow diffusion with high free energy barriers reaching up to ∼20 [*k*_B_ *T*]. Most MOFs with slow fixed water-hindered diffusion under dilute conditions show high 1D free energy barriers above 6 [*k*_B_ *T*], except for PAMVEG, which has barriers smaller than 3 [*k*_B_ *T*], as shown in Figure 7l. These results suggest that, in general, 1D free energy barriers can be used to infer the Ds of water under dilute conditions. Furthermore, we identify the spatial distribution of favorable adsorption sites for water molecules (i.e., those with energy <−4500 K) in these MOFs following the method developed by Xu et al.^55^ The threshold of −4500 K is based on the criteria for favorable adsorption sites defined in their work. Figure S4a–h reveal that MOFs exhibiting fast unconstrained water diffusion have no favorable adsorption sites. In contrast, MOFs exhibiting slow diffusion under dilute conditions, characterized by strong adsorption-shielded diffusion and fixed water-hindered diffusion, have multiple favorable adsorption sites, as shown in Figure S4i–n. These sites strongly adsorb water molecules in MOFs, resulting in slow diffusion.

**Figure 7:**
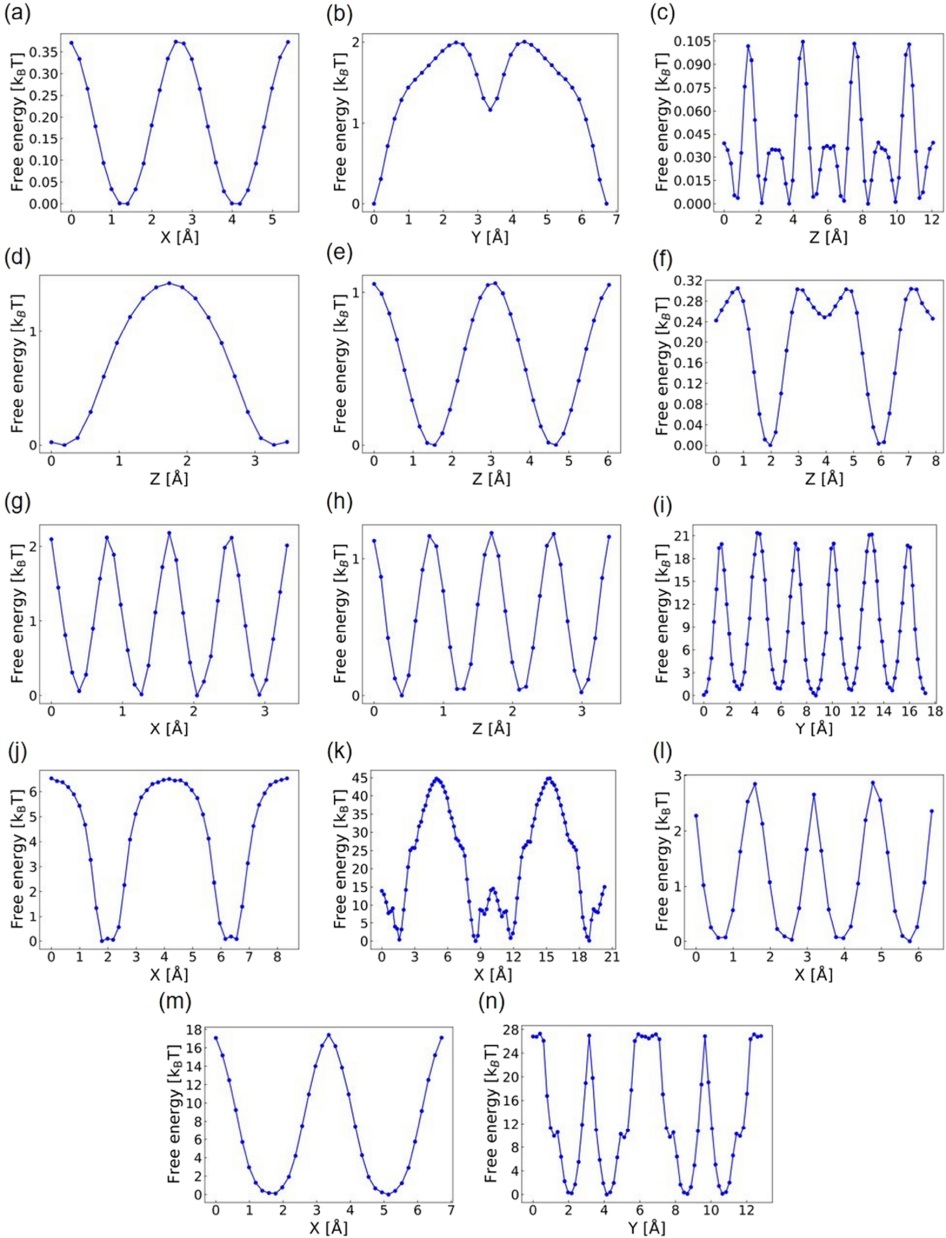
1D free energy plots along each diffusion channel for various MOFs probed under dilute conditions, including: (a) PEKZIP, (b) LUFQUZ, (c) KUZPEC, (d) KOGYIP, (e) OHIHET, (f) NAVLIG, (g) MAXHOJ, (h) CUGYOU, (i) FULQUZ, (j) EQUBOI, (k) DUYREV, (l) PAMVEG, (m) FUWYAY, and (n) DATHAJ.

To further explore this, we focus on two MOFs—FULQUZ and PAMVEG—that exhibit strong adsorption-shielded diffusion and fixed water-hindered diffusion, respectively. Both MOFs show slow diffusion under dilute conditions. We analyze the 1D and 2D free energy plots of these MOFs under both dilute and saturated conditions, with the results summarized in Figure 8. Since all adsorption sites are occupied under saturated conditions, we approximate the free energy plots for these conditions using a unit cell instead of a supercell. This approach allows for better comparisons with the free energy plots calculated under dilute conditions. As shown in Figure 8a,b, the 1D free energy plots under saturated conditions indicate small free energy barriers for both FULQUZ and PAMVEG. This accurately reflects the fast diffusivity of FULQUZ under saturated conditions but incorrectly suggests fast diffusivity for PAMVEG, which actually diffuses slowly. Figure 8c,d show that the 2D free energy profiles under dilute conditions have large barriers along the diffusion channels for both MOFs, correctly reflecting their slow diffusion. Under saturated conditions, the 2D landscapes displayed in Figure 8e,f show small barriers for FULQUZ and large barriers for PAMVEG along their diffusion channels, accurately indicating their respective diffusivities. Interestingly, when the 2D free energy plot of PAMVEG is projected onto 1D, the overlay of adsorption sites upon projection reduces the perceived barrier. Based on these findings, we conclude that 1D free energy barriers may infer water diffusivity provided that adsorption sites are not overlaid upon projection. However, 2D free energy landscapes, whether under dilute or saturated conditions, provide a more accurate reflection of diffusivities under various conditions.

**Figure 8:**
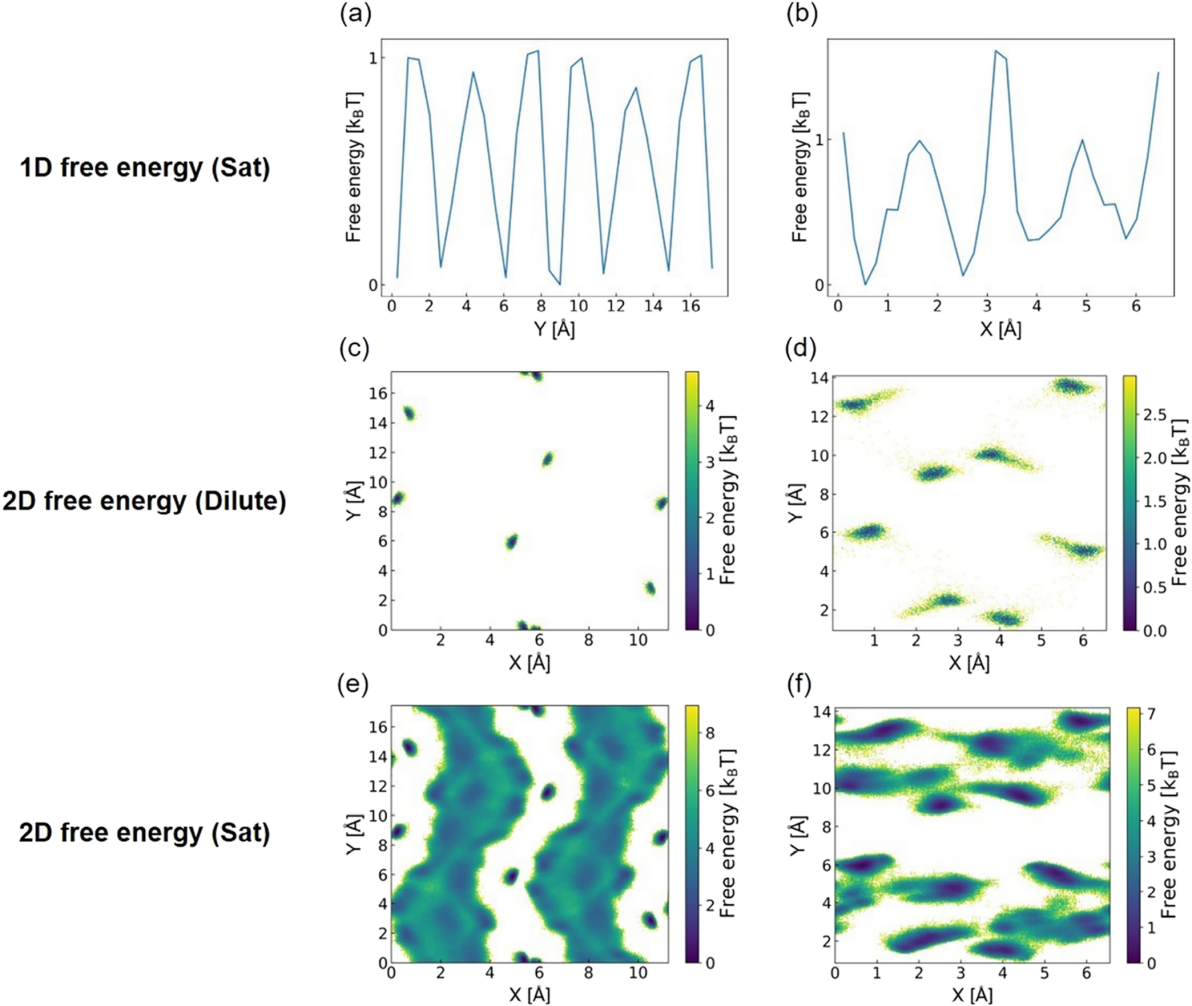
1D and 2D free energy plots along each diffusion channel for various MOFs under different conditions: (a) 1D free energy plot for FULQUZ under saturated conditions, (b) 1D free energy plot for PAMVEG under saturated conditions, (c) 2D free energy plot for FULQUZ under dilute conditions, (d) 2D free energy plot for PAMVEG under dilute conditions, (e) 2D free energy plot for FULQUZ under saturated conditions, and (f) 2D free energy plot for PAMVEG under saturated conditions.

Based on the conclusions drawn from Figure 8, which shows that 2D free energy plots offer better inferability of diffusivities, we further evaluate the water diffusivity in MOFs with varying diffusion mechanisms using these 2D plots, as shown in Figure 9. Figure 9a depicts the 2D free energy plot of an example MOF (OHIHET) exhibiting fast unconstrained water diffusion, characterized by low free energy barriers and a homogeneous free energy landscape due to its unconstrained energies and topologies. It is noted that due to the MOF’s highly hydrophobic nature, most water molecules tend to desorb at saturated pressure during the desorption process. To address this, we fill the pores with water molecules to mimic full saturation. Given that this MOF has a total of 8 channels in a supercell, with each channel capable of containing 9 water molecules, a total water loading of 72 was used to represent fully saturated conditions, rather than the calculated saturated water loading of 18 to simulate fully saturated conditions. Interestingly, despite this increased loading, the Ds values for *N* = 18 (4.78 × 10^–5^ cm^2^/s) and *N* = 72 (2.39 × 10^–5^ cm^2^/s) are quite similar. Figure 9b presents the 2D free energy plot of an example MOF (XUMSOP), which exhibits slow topological bottleneck-hindered diffusion. The small PLD, highlighted by a pink circle, creates a diffusion bottleneck. This leads to a heterogeneous free energy landscape with high free energy barriers along the diffusion path, accurately reflecting the slow diffusion observed in this MOF. Figure 9c shows the 2D free energy plot of an example MOF (FULQUZ) with strong adsorption-shielded diffusion. In the plot, water molecules are fixed along the diffusion channel, creating a shielding effect. This effect leads to a relatively uniform free energy landscape with small free energy barriers along the diffusion path, which aligns with the observation of fast diffusion in this MOF. Lastly, Figure 9d illustrates the 2D free energy plot of an example MOF (FUWYAY) with fixed water-hindered diffusion. In this plot, the fixed water molecules along the diffusion channels reduce the pore size and obstruct the passage of additional water molecules, consequently creating high free energy barriers along the diffusion channel. This correctly reflects the observed slow water diffusion.

**Figure 9:**
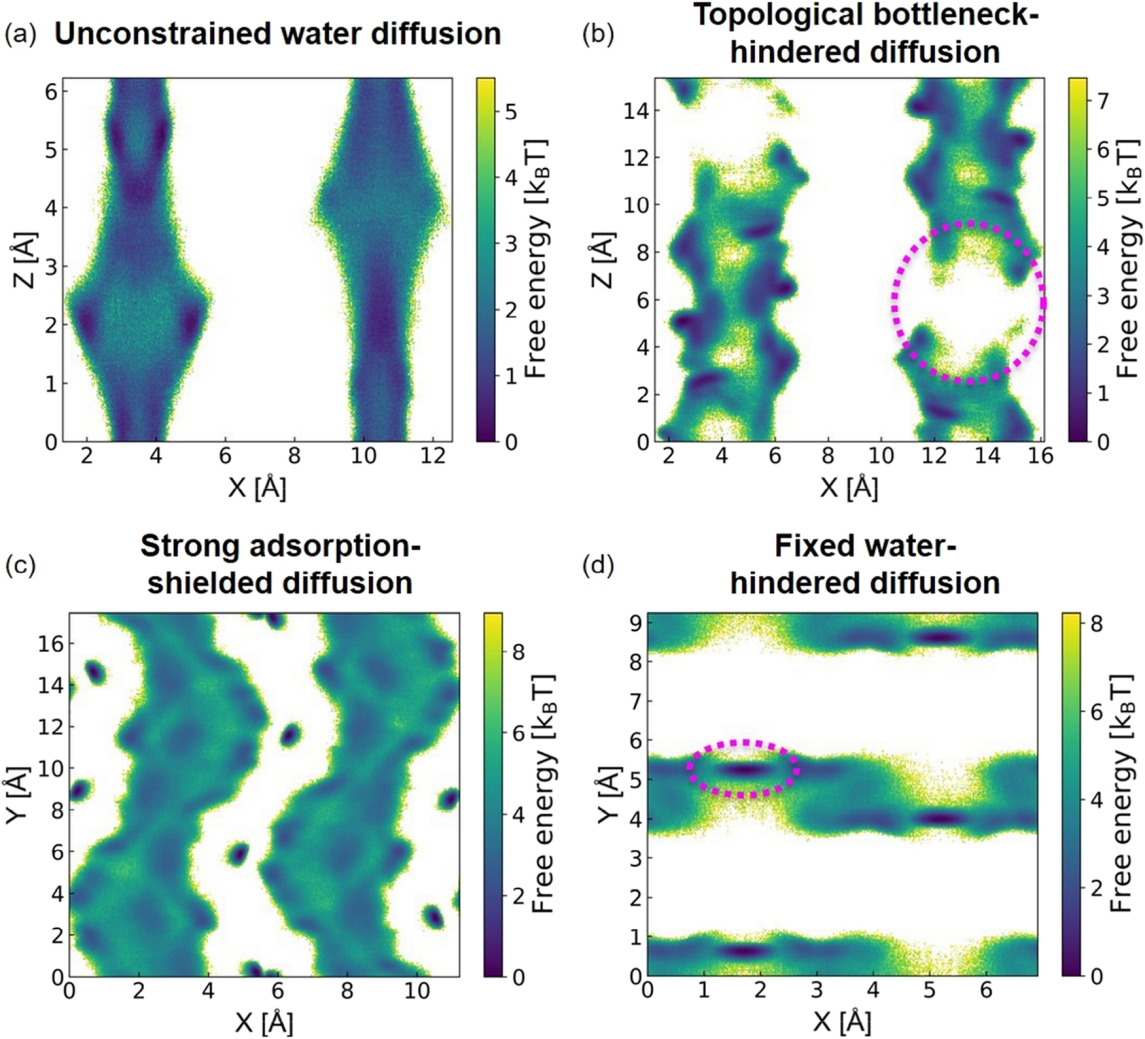
2D free energy plots under saturated conditions for: (a) example MOF (OHIHET) with unconstrained water diffusion, (b) example MOF (XUMSOP) with topological bottleneck-hindered diffusion, (c) example MOF (FULQUZ) with strong adsorption-shielded diffusion, and (d) example MOF (FUWYAY) with fixed water-hindered diffusion. The topological bottleneck and the bottleneck due to the presence of fixed water are circled in pink.

## 4 Conclusions

In this study, we conduct a large-scale screening to identify MOFs with rapid water diffusion and uncover key factors that impact water diffusivity. Our findings indicate that water diffusivity under saturated conditions is primarily influenced by the strength of interactions between water molecules and metal sites, the pore size of the MOFs, and, importantly, their interplay. Interestingly, we observe varied diffusion behaviors among MOFs with small pores and similar framework-water interactions due to diverse diffusion mechanisms. Specifically, in MOFs with weak framework-water interactions, ultrafast single-file diffusion occurs in those with small pores, while slower diffusion is seen in MOFs with even smaller pores. Conversely, in MOFs with strong framework-water interactions, the presence of fixed water induces a shielding effect and acts as a physical barrier, leading to varied diffusivities based on pore size. Finally, the comparison between dilute and saturated conditions reveals that diffusivity under dilute conditions can be directly used to infer those under saturated conditions, provided that it does not involve strong adsorption-shielded diffusion. This study also finds that 1D free energy profiles may be inadequate for accurately capturing transport properties under saturated conditions, highlighting the need for 2D free energy profiles for more precise analysis. These insights are crucial for the development of MOFs for water-related applications, paving the way for more effective utilization of these materials in water harvesting and filtration technologies.

## Supplementary Material









## Data Availability

Most electronic Supporting Information files are available without a subscription to ACS Web Editions. Such files may be downloaded by the article for research use (if there is a public use license linked to the relevant article, that license may permit other uses). Permission may be obtained from ACS for other uses through requests via the Rights Link permission system: http://pubs.acs.org/page/copyright/permissions.html

## Notes

Simulation details of free energy landscape, additional tables referred in the main text including force field parameters and characteristics of water diffusion in selected MOFs, and additional figures referred in the main text including correlations between diffusivity and key geometric and chemical descriptors, time evolution of water-metal distance in FULQUZ, 2D free energy profiles of MOFs with topological bottleneck-hindered diffusion, and spatial distribution of favorable water adsorption sites in selected MOFs (PDF)
Diffusion properties and features of all studied MOFs (XLSX)
Movie S1: Fixed water-hindered diffusion mechanism (AVI)
Movie S2: Strong adsorption-shielded diffusion (AVI)
