# Supplementary material for: Large-scale Investigations of Water Diffusion in Metal–Organic Frameworks with One-Dimensional Channels
Source: ACS Appl Mater Interfaces. 2025 Sep 3;17(37):52223–35. doi: 10.1021/acsami.5c12959 (PMC12447398; doi:10.1021/acsami.5c12959)
Supplement: Supplementary file 1 [file am5c12959_si_001.pdf]

Supporting Information

for

Large-Scale Investigations of Water Diffusion in Metal-Organic Frameworks with One-Dimensional Channels

I-Ting Sung[a] and Li-Chiang Lin[a,b,]*

[a]*Department of Chemical Engineering, National Taiwan University, No. 1, Sec. 4, Roosevelt Road, Taipei 106319, Taiwan*

[b]*William G. Lowrie Department of Chemical and Biomolecular Engineering, The Ohio State University, 151 W. Woodruff Avenue, Columbus, OH 43210, United States.*

*E-mail: *lclin@ntu.edu.tw*

1. Simulation details

1.1 Free energy landscape under saturated conditions

Free energy landscapes are crucial for understanding the diffusion rate of water molecules within MOFs. To explore these landscapes, Monte Carlo simulations are conducted in a canonical ensemble (NVT) at saturated conditions. These simulations maintain a constant temperature of 298 K and involve sampling millions of molecular configurations through equal proportions of translation, rotation, and reinsertion movements. Typically, equilibration cycles are 10,000 followed by production cycles of 200,000 per simulation. This approach allows for the relative probability of sampling the oxygen atoms in water molecules at a cross-section perpendicular to the flow to be calculated. This data is essential for mapping out the free energy landscape. The formula used to compute the free energy is displayed below:

$$F = -\ln(P_q/P_{\mathrm{ref}})\ [k_{\mathrm{B}}T] \tag{1}$$

Where F represents the free energy, with k_{B} and T corresponding to the Boltzmann constant and the temperature, respectively. The P_q denotes the likelihood of locating an oxygen atom of water at a given cross-section q. The reference position is defined as the point where the energy reaches its minimum value.

1.2 Free energy landscape under dilute conditions

To construct the free energy landscape under dilute conditions, we use a method adapted from the work of Lin and coworkers[1]. Specifically, the Widom insertion method is applied at 298 K for a minimum of 2,000,000 cycles to ensure convergence. This method samples the adsorption energy for each configuration inserted into the MOF under infinite dilution conditions. The position of the water's oxygen atom is chosen as a representative location. To enhance sampling, each inserted configuration is folded back into the unit cell of the MOF. This unit cell is then divided into grids, with the grid size set to 0.2 Å in this study. For each grid, the free energy F is computed by taking the negative natural logarithm of the sum of Boltzmann factors for all sampled configurations. The formula used to compute the free energy is displayed below:

$$F = -\ln \sum_{i=1}^{N} \mathrm{e}^{\frac{-\Delta E_i}{T}}\ [k_{\mathrm{B}}T] \tag{2}$$

2. Tables referred in the main text

Table S1. Lennard-Jones parameters for the atoms of MOFs

Atom type [-]	ε/k_B [K]	σ [Å]
Ag	18.11	2.80
Al	156.08	3.91
B	47.83	3.58
Ba	183.15	3.30
Br	186.29	3.52
C	47.88	3.47
Ca	119.75	3.03
Cd	114.72	2.54
Ce	6.54	3.17
Cl	142.64	3.52
Co	7.04	2.56
Cr	7.55	2.69
Cu	2.52	3.11
Dy	3.52	3.05
Er	3.52	3.02
F	36.50	3.09

Fe	6.54	2.59
Ga	208.81	3.90
Gd	4.53	3.00
Ge	190.69	3.81
H	7.65	2.85
Ho	3.52	3.04
I	256.77	3.70
In	276.91	4.09
K	17.61	3.40
La	8.55	3.14
Li	12.58	2.18
Lu	20.63	3.24
Mg	55.85	2.69
Mn	6.54	2.64
N	38.97	3.26
Na	15.11	2.66
Nd	5.03	3.18
Ni	7.55	2.52
O	48.18	3.03

P	161.11	3.70
Pd	24.15	2.58
Pr	5.03	3.21
Pt	40.25	2.45
S	173.20	3.59
Sc	9.56	2.94
Si	156.08	3.80
Sm	4.03	3.14
Sr	118.24	3.24
Tb	3.52	3.07
Tm	3.02	3.01
U	11.07	3.02
V	8.05	2.80
W	33.71	2.73
Y	36.23	2.98
Yb	114.72	2.99
Zn	27.69	4.04
Eu	3.99	3.11

Table S2. Lennard-Jones parameters and partial charges of the TIP4P-EW water model. The atom type M is the massless site of the TIP4P-EW water model.

Atom type [-]	ε/k_B [K]	σ [Å]	q [-]
O	81.92	3.16	0.000
H	0.00	0.00	0.524
M	0.00	0.00	-1.048

Table S3. Characteristics of water diffusion in the studied MOFs, including details on their chemical formulas, diffusion mechanisms, HOA values, and PLD values. Note that chemical formulas are taken from the literature and may include solvents; for all simulations, guest solvents were removed during structure curation. Each MOF is identified by its refcode, which can be cross-referenced in the CSD database[2] and MOFX-DB[3] to retrieve the source publication and any common name.

Refcode	Chemical formula	Diffusion mechanism	HOA [kJ/mol]	PLD [Å]
KOGYIP	$(C_{20}H_{16}Cl\,Cu_2N_4^{+})_{n,n}(NO_3^{-})$	Unconstrained	-17.72	4.86
LUFQUZ	$(C_8H_4Fe\,N_6Ni)_n,2n(H_2O)$	Unconstrained	-24.42	3.71
NAVLIG	$(C_{48}H_{40}Cu_2N_8^{2+})_{n,2n}(NO_3^{-})$	Unconstrained	-17.66	3.74
OHIHET	$(C_{24}H_{20}N_8Zn)_n,4n(H_2O)$	Unconstrained	-14.96	3.71
PEKZIP	$(C_{20}H_{16}Cl\,Cu_2N_4^{+})_{n,n}(Cl\,O_4^{-})$	Unconstrained	-14.25	4.09
KUZPEC	$(C_{24}H_{24}Ag\,N_4^{+})_{n,n}(C\,F_3O_3S^{-}),H_2O$	Unconstrained	-12.68	3.98
CUGYOU	$(C_8H_{12}Ag_6Br\,N_{16}^{+})_n,6n(H_2O),_n(HO^{-})$	Unconstrained	-22.81	7.98
MAXHOJ	$(C_8H_{12}Ag_6Cl\,N_{16}^{+})_{n,n}(HO^{-}),6n(H_2O)$	Unconstrained	-31.41	7.15
FULQUZ	$(C_{24}H_{22}La_2N_2O_{15}S_3)_{n,n}(C_3H_7N\,O)$	Strong adsorption-shielded	-91.70	5.25
DATHAJ	$(C_{21}H_{27}N_4Nd_3O_{23})_n,5n(H_2O)$	Fixed water-hindered	-138.05	3.71
EQUBOI	$(C_{14}H_{14}Eu_2O_{14}S_2)_n,5n(H_2O)$	Fixed water-hindered	-53.11	4.12
FUWYAY	$(C\,H_5Mn_2N_5O_6S)_n,0.25n(H_2O)$	Fixed water-hindered	-94.11	3.87
PAMVEG	$(C_{16}H_{22}N_4O_4Zn)_n,3n(H_2O)$	Fixed water-hindered	-41.63	3.71
DUYREV	$(C_{21}H_{34}N_3O_{62}Pr_3W_{12})_n,10n(H_2O)$	Fixed water-hindered	-178.52	3.70
TUKYIJ	$(C_{30}H_{42}Ag_2N_{12}^{2+})_{n,n}(C_4H_2O_4^{2-})$	Topological bottleneck-hindered	-24.36	2.80
PULHOT	$(C_6H_{14}N_2^{2+})_{n,n}(H_2Ga\,Mn\,O_{12}P_3^{2-})$	Topological bottleneck-hindered	-63.63	2.80
IZEVAM	$(C_6H_{12}Cl_6Cu_4N_4^{2-})_{n,n}(C_6H_{13}Cu_3N_4O^{2+}),3n(H_2O)$	Topological bottleneck-hindered	-22.28	2.81

NIGLUK	$(C_{28}\,H_{16}\,Cu\,N_4^{+})_{n,n}(F_6\,P^{-})$	Topological bottleneck-hindered	-18.65	2.80
XUMSOP	$(C_{36}\,H_{48}\,Ho_4\,O_{24})_n$	Topological bottleneck-hindered	-37.66	2.81

3. Figures referred in the main text

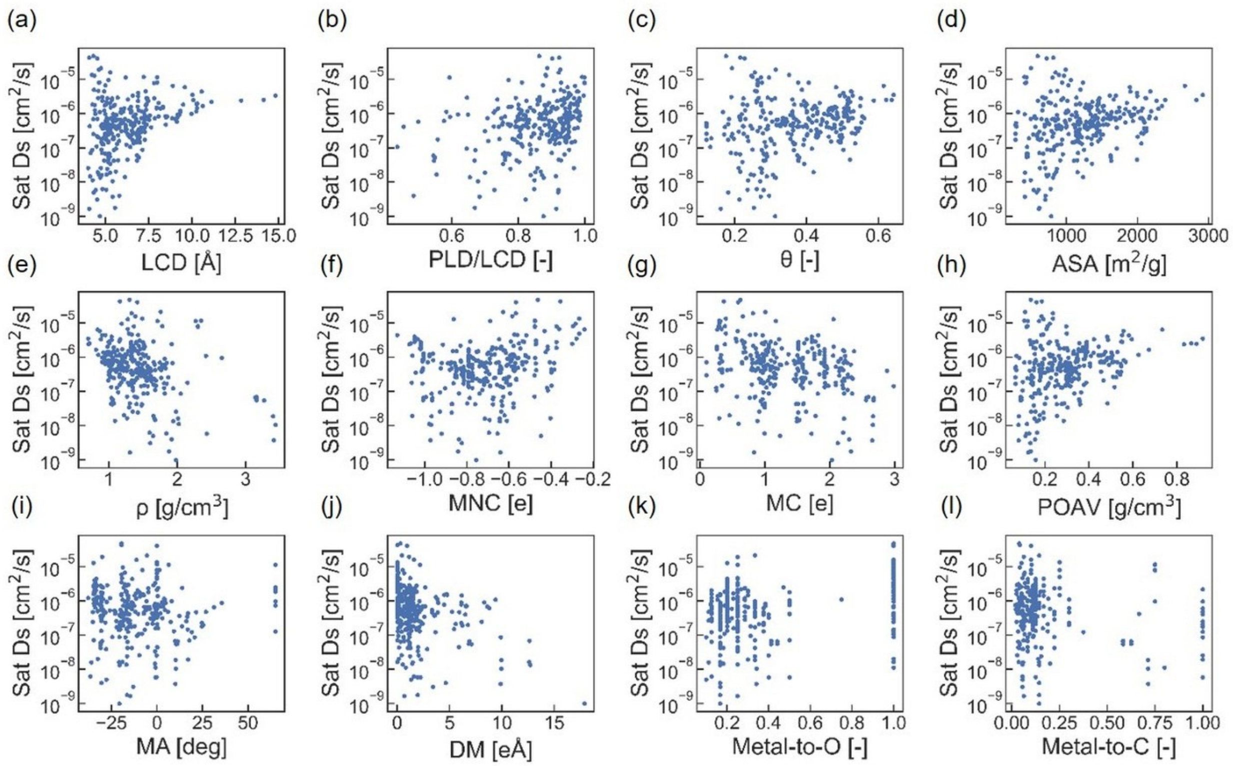

Figure S1. Large-scale screening results of the relation of Sat Ds and other features, including (a) largest cavity diameter (LCD), (b) PLD-to-LCD ratio (PLD/LCD), (c) void fraction (θ), (d) accessible surface area (ASA), (e) density (ρ), (f) most negative charge (MNC), (g) maximum charge (MC), (h) probe occupiable and accessible pore volume (POAV), (i) metal angle (MA), (j) dipole moment (DM), (k) metal-to-oxygen ratio (Metal-to-O), and (l) metal-to-carbon ratio (Metal-to-C).

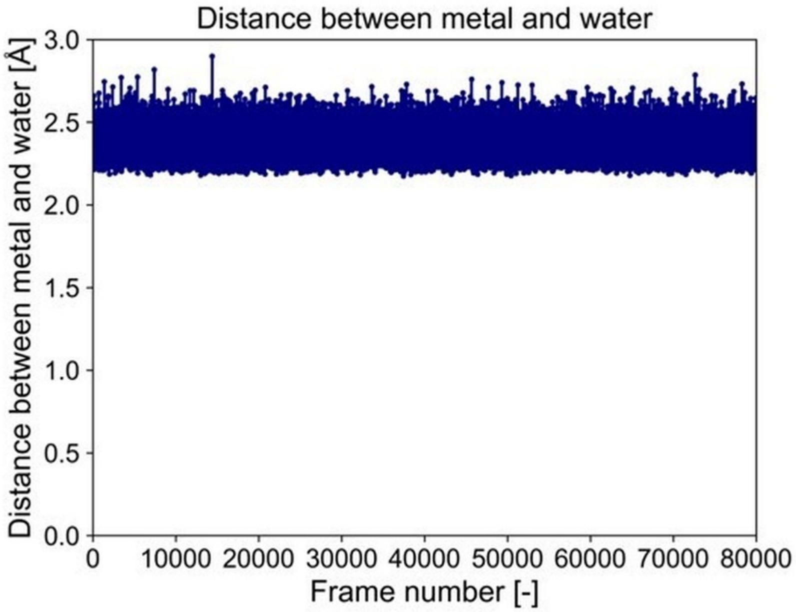

Figure S2. The distance between the metal of FULQUZ and the fixed water recorded every 1000 time steps (1 picosecond).

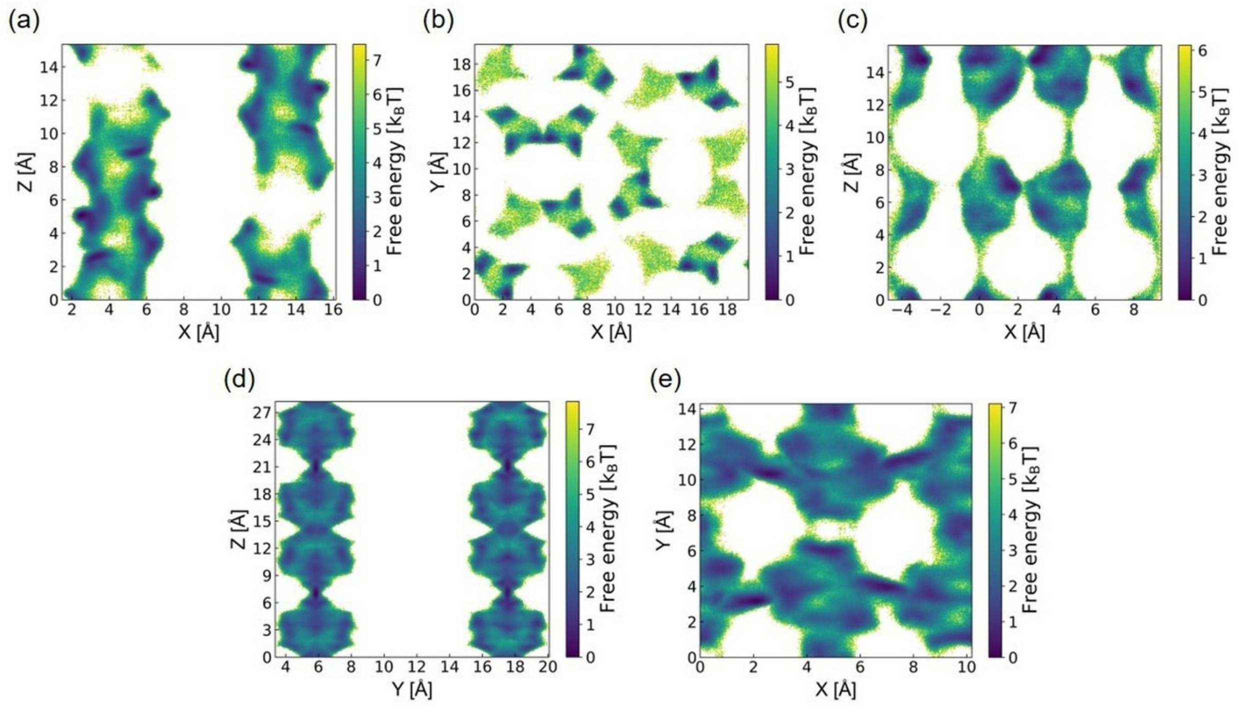

Figure S3. 2D free energy plots of MOFs with topological bottleneck-hindered diffusion, including (a) XUMSOP, (b) NIGLUK, (c) IZEVAM, (d) TUKYIJ, and (e) PULHOT.

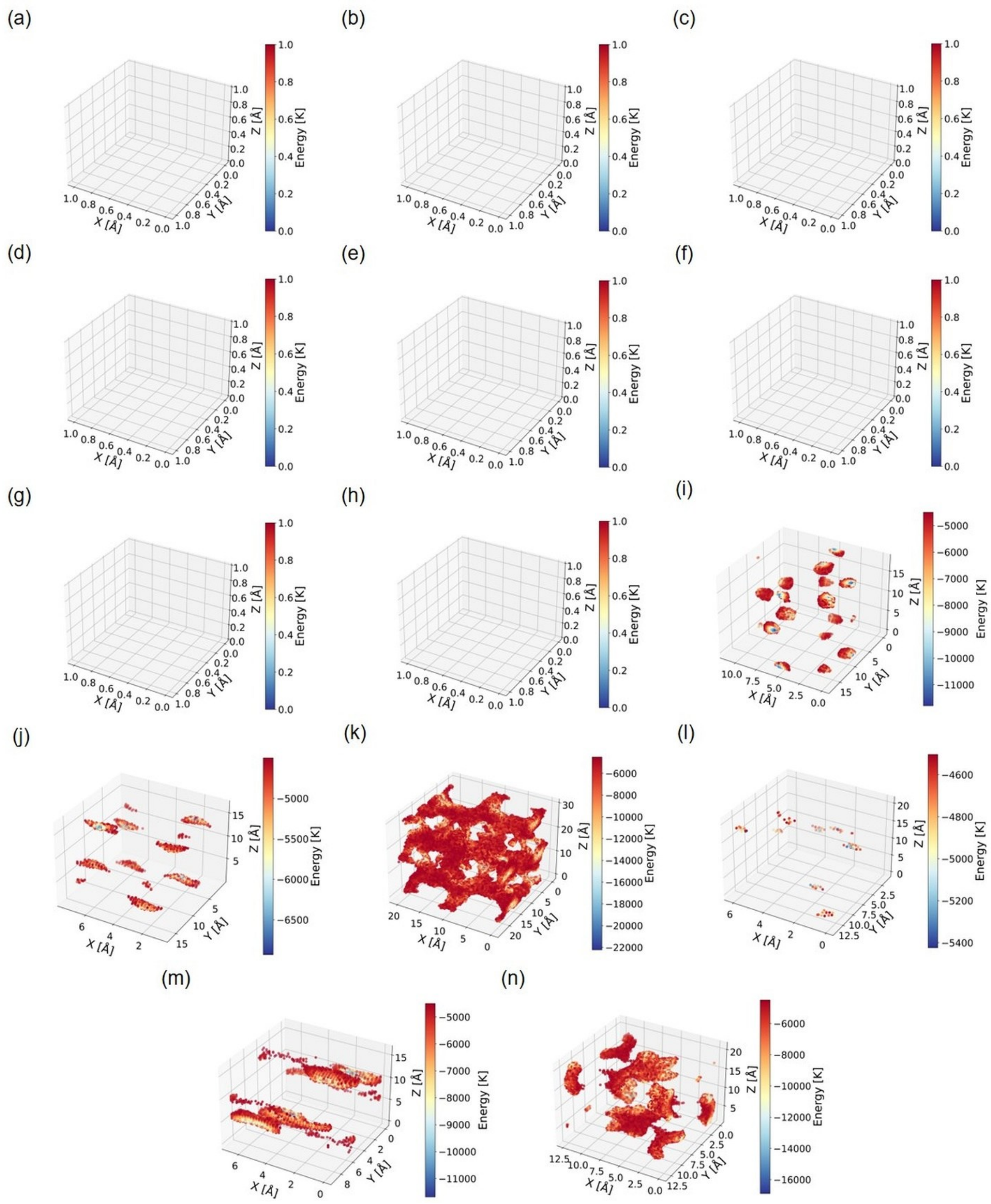

Figure S4. Spatial distribution of favorable water adsorption sites (i.e., those with energy < -4500 K) in (a) PEKZIP, (b) LUFQUZ, (c) KUZPEC, (d) KOGYIP, (e) OHIHET, (f) NAVLIG, (g) MAXHOJ, (h) CUGYOU, (i) FULQUZ, (j) EQUBOI, (k) DUYREV, (l) PAMVEG, (m)

FUWYAY, and (n) DATHAJ. It is noted that, for (a-h), no adsorption sites with energy lower than -4500 K are identified in the structure.

5. References

(1) Xu, Z.-X.; Wang, Y.-M.; Lin, L.-C. Connectivity Analysis of Adsorption Sites in Metal–Organic Frameworks for Facilitated Water Adsorption. *ACS Appl. Mater. Interfaces* **2023**, *15* (40), 47081–47093. https://doi.org/10.1021/acsami.3c10710.

(2) Groom, C. R.; Bruno, I. J.; Lightfoot, M. P.; Ward, S. C. The Cambridge Structural Database. Acta Crystallogr. Sect. B Struct. Sci. Cryst. Eng. Mater. 2016, 72 (2), 171–179. https://doi.org/10.1107/S2052520616003954.

(3) Bobbitt, N. S.; Shi, K.; Bucior, B. J.; Chen, H.; Tracy-Amoroso, N.; Li, Z.; Sun, Y.; Merlin, J. H.; Siepmann, J. I.; Siderius, D. W.; Snurr, R. Q. MOFX-DB: An Online Database of Computational Adsorption Data for Nanoporous Materials. J. Chem. Eng. Data 2023, 68 (2), 483–498. https://doi.org/10.1021/acs.jced.2c00583.
